# Changes in plasma albumin levels in early detection of infectious complications after laparoscopic colorectal cancer surgery with ERAS protocol

**DOI:** 10.1007/s00464-018-6040-4

**Published:** 2018-01-16

**Authors:** Mateusz Wierdak, Magdalena Pisarska, Beata Kuśnierz-Cabala, Jan Witowski, Jadwiga Dworak, Piotr Major, Piotr Małczak, Piotr Ceranowicz, Andrzej Budzyński, Michał Pędziwiatr

**Affiliations:** 1 2nd Department of General Surgery, Jagiellonian University Medical College, Kopernika 21, 31-501 Kraków, Poland; 2 Centre for Research, Training and Innovation in Surgery (CERTAIN Surgery), Kraków, Poland; 3 Department of Diagnostics, Chair of Clinical Biochemistry, Jagiellonian University Medical College, Kraków, Poland; 4 Department of Physiology, Jagiellonian University Medical College, Kraków, Poland

**Keywords:** Colorectal cancer, Laparoscopy, Infectious complications, Markers, Albumins

## Abstract

**Background:**

Combination of laparoscopic approach with ERAS protocol in colorectal surgery allows for an early discharge. However there is a risk that some of the discharged patients are developing, asymptomatic at the time, infectious complications. This may lead to a delay in diagnostics and proper treatment introduction. We aimed to assess the usefulness of preoperative plasma albumin concentration and their changes as indicators of infectious complications in patients undergoing colorectal cancer surgery.

**Methods:**

Prospective analysis included 105 consecutive patients who underwent laparoscopic colorectal cancer resection between August 2014 and September 2016. In all cases standardised 16-item perioperative care ERAS protocol was used (mean compliance > 85%). Patients with IBD, distant metastases, undergoing emergency or multivisceral resection were excluded. Blood samples were collected preoperatively and on POD 1, 2, 3. Plasma albumin concentration was measured. Patients were divided into two groups depending on the presence of infectious complications. We analysed the differences in the levels of albumin and the dynamics of changes.

**Results:**

Group 1—82 not complicated patients, Group 2—23 patients with at least one infectious complication. Preoperatively, there were no significant differences in the levels of serum albumin between those groups (Group 1—38.7 ± 4.9 g/l; Group 2—37.7 ± 5.0 g/l). In postoperative period, decrease was observed in both (POD 1: Group 1—36.5 ± 4.2 g/l, Group 2—34.7 ± 4.2 g/l, *p* = 0.07; POD 2: Group 1—36.2 ± 4.1 g/l, Group 2—32.6 ± 5.6 g/l, *p* = 0.01; POD 3: Group 1—36.0 ± 4.4 g/l, Group 2—30.9 ± 3.5 g/l, *p* = 0.01). The decrease was significantly greater in Group 2 on POD 2 and 3.

**Conclusions:**

We showed that a regular measurement of albumin in the early postoperative days may be beneficial in the detection of postoperative infectious complications. Although changes in albumins are observed early after surgery, this parameter is relatively unspecific.

Colorectal cancer surgery is associated with a relatively high morbidity rate, which depends on multiple factors and can occur in 30–40% of patients [1, 2]. One of the significant components affecting those parameters is the surgical technique. It has been shown that the laparoscopic approach is correlated with reduced morbidity [3, 4]. Moreover, the implementation of multimodal perioperative care protocols enhanced recovery after surgery (ERAS) further decreases postoperative complications by approximately 20–40% and leads to early hospital discharge after 2–5 days [5–8]. These factors markedly shortened length of stay (LOS). Some complications (i.e. anastomotic leakage) can occur late after surgery, even on postoperative day (POD) 8–12. This means that in most cases they are diagnosed after discharge [9]. For these reasons, markers that allow early detection of complications and prediction of their severity, ideally before patients become symptomatic, are the area of interest for many surgeons.

Albumin, which is mostly used as a nutritional marker and a predictor for outcomes, is a protein which immediately responds to surgical stress. An albumin drop is observed in most major abdominal surgeries within the first postoperative hours [10–12]. Although pathophysiological basics of albumin kinetics are well-established, this parameter is rarely used as a marker of complications in the early postoperative period. Also, the albumin level correlates with the surgical trauma and postoperative stress response [13]. It is important in the context of laparoscopy and ERAS protocol, since they both significantly reduce the degree of surgical stress [14, 15]. That is why it seems reasonable to establish whether regular assessments of albumin level in the early postoperative phase are clinically relevant.

## Aim

The aim of this study was to evaluate the usefulness of albumin level measurements as an early predictor of infectious complications in patients with colorectal cancer undergoing laparoscopic surgery with ERAS protocol.

## Materials and methods

The study was conducted in a tertiary referral centre (university hospital). Consecutive patients undergoing laparoscopic resection for colorectal cancer were prospectively analysed. Inclusion criteria were age over 18 years old, elective laparoscopic surgery for verified colorectal adenocarcinoma and ERAS protocol in perioperative care. We excluded patients who underwent open or emergency surgery and those in which resection exceeding the large bowel (T4) was required. Patients with inflammatory bowel disease were excluded from the study as well as cases where it was not possible to implement ERAS protocol (i.e. due to hospitalisation in ICU). Other exclusion criteria included distant metastases (M1), rectal cancer treated with transanal endoscopic microsurgery, conversion to open resection, patients with an active infection or autoimmune systemic disease. Moreover, we excluded patients in whom infectious complications were diagnosed within the first 48 h postoperatively.

A laparoscopic approach with five or six trocars and medial to lateral technique was used [16]. All patients had the exact same perioperative care ERAS protocol (Table 1), which has been used in our department for 5 years. Mean compliance with the protocol is over 80% [17].

**Table 1:** ERAS protocol used in our unit

1 Preoperative counselling and patient’s education
2 No bowel preparation (oral lavage in the case of low rectal resection with TME and defunctioning loop ileostomy)
3 Preoperative carbohydrate loading (400 ml of Nutricia preOp® 2 h prior surgery)
4 Antithrombotic prophylaxis (Clexane® 40 mg sc. starting in the evening prior surgery)
5 Antibiotic prophylaxis (preoperative cefuroxime 1.5 g + metronidazole 0.5 g iv. 30–60 min prior surgery)
6 Laparoscopic surgery
7 Balanced intravenous fluid therapy (< 2500 ml intravenous fluids during the day of surgery, less than 150 mmol sodium)
8 No nasogastric tubes postoperatively
9 No drains left routinely for colonic resections, one drain placed for < 24 h in case of TME
10 Transversus abdominis plane (TAP) block, epidural anaesthesia in cases with high risk of conversion
11 Avoiding opioids, multimodal analgesia (oral when possible—paracetamol 4 × 1 g, ibuprofen 2 × 200 mg, metamizole 2 × 2.5 g, or ketoprofen 2 × 100 mg)
12 Prevention of postoperative nausea and vomiting (PONV) (dexamethasone 8 mg iv., ondansetron 8 mg iv., metoclopramide 10 mg iv.)
13 Postoperative oxygenation therapy (4–6 l/min)
14 Early oral feeding (oral nutritional supplement 4 h postoperatively, light hospital diet and oral nutritional supplements on the first postoperative day, full hospital diet on the second postoperative day)
15 Urinary catheter removal on the first postoperative day
16 Full mobilisation on the first postoperative day (getting out of bed, going to toilet, walking along the corridor, at least 4 h out of bed)

Blood samples for albumin measurements were drawn from the patients four times: on the day of surgery and on the three following PODs. Albumin measurements were included in the routine biochemistry panel; they were performed in serum on the day of blood collection using the automated analyser Cobas 6000 (Roche Diagnostics, Basel, Switzerland).

Serum samples were obtained by centrifuging blood, collected without anticoagulant, for 10 min at 4000 rpm on a MPW 351e centrifuge (MPW Med. Instruments, Warsaw, Poland; Rotor No. 12,436). The reference interval for albumin was 35–50 g/l.

Patients were divided into Groups 1 and 2 that included patients without and with infectious complications, respectively. The diagnosis of these complications and assessment of their severity was performed according to ECDC guidelines [18].

Groups were compared for age, sex, body mass index (BMI), American Society of Anaesthesiologists (ASA) score, type of surgery, tumour staging, operative time and intraoperative blood loss. Differences in albumin level between groups were analysed on consecutive PODs. Due to the high variability in preoperative measurements, Δ-increments were calculated each day (i.e. ΔPOD1: difference of concentration on POD1 and POD0; ΔPOD2: difference of concentration on POD2 and POD0 etc). Additionally, albumin level ratios were calculated (i.e. POD1/0, POD 3/0, POD 3/1 etc.).

## Statistical analysis

All data were analysed with Statsoft STATISTICA v.12. The results are presented as mean ± standard deviation (SD), median and interquartile range (IQR). The study of categorical variables used the Chi-square test of independence. The Shapiro–Wilk test was used to check for a normal distribution of data and the Student t test was used for normally distributed quantitative data. For non-normally distributed quantitative variables, the Mann–Whitney U test was used. For dependent variables the Friedman test was used. A receiver operating characteristic (ROC) curve was applied to obtain the area under the curve (AUC) and determine the best cut-offs. Results were considered statistically significant when p value was less than 0.05.

The study was approved by the local Ethics Review Committee (Approval Number KBET/211/B/2014). All procedures were performed in accordance with the ethical standards laid down in the 1964 Declaration of Helsinki and its later amendments.

## Results

**Fig. 1:**
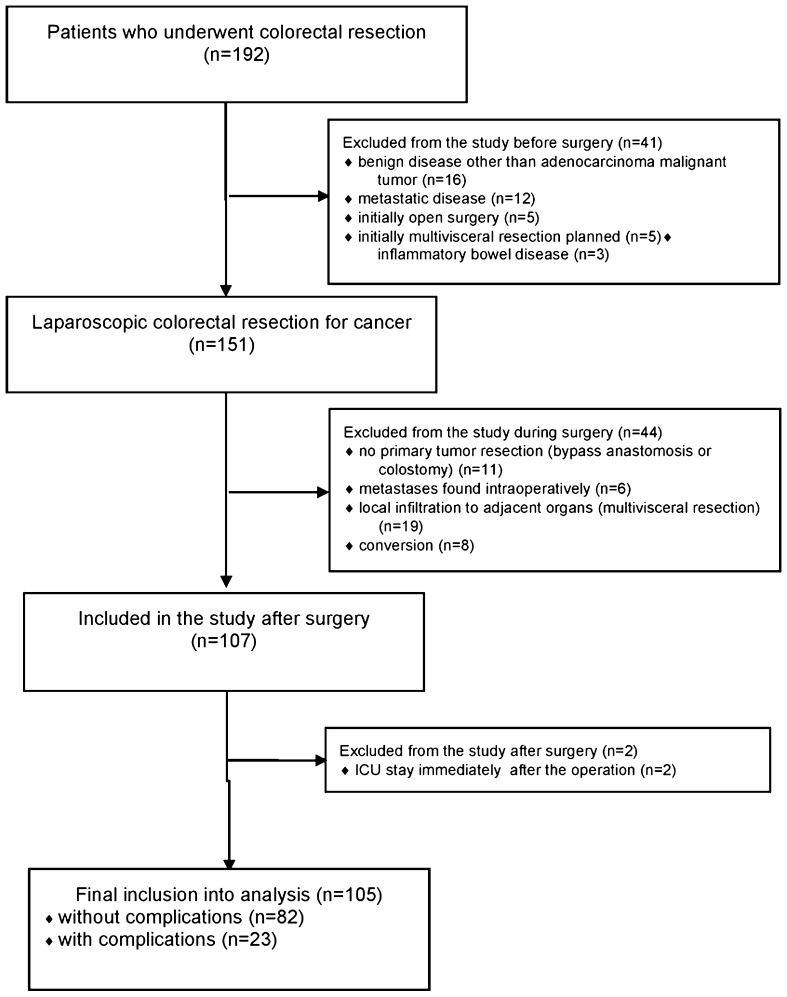
Patients flow through the study

192 patients in our department underwent colorectal resection between August 2014 and September 2016. 41 of them did not fulfil inclusion criteria and were initially excluded. 44 were excluded during surgery. Two patients were excluded because ERAS protocol was not implemented in the postoperative period. Patients’ flow through the study and reasons for exclusion are shown in Fig. 1.

Groups 1 and 2 consisted of 82 (78.1%) and 23 (21.9%) patients, respectively. Table 2 shows a demographic analysis of groups. No significant differences were noticed regarding their demography, ASA scale, type of performed surgery, operative time, intraoperative blood loss and cancer stage. However, there were significant differences between the groups in median LOS (4 vs. 9 days, *p* < 0.001) and readmission rate (4.9 vs. 17.4%, *p* = 0.046). In addition, we did not notice differences in mean intravenous fluids transfused during the surgical procedure and first 24 h postoperatively (2134 ± 665 vs. 2333 ± 901 ml, *p* = 0.42). The compliance with restrictive intravenous fluid therapy according to ERAS protocol was 91.4 vs. 87.1% in Group 1 and 2, respectively (*p* = 0.51).

**Table 2:** Demographic analysis of patient groups

Parameter	Group 1 (uncomplicated)	Group 2 (complicated)	*p* value
Number of patients [n (%)]	82 (78.1%)	23 (21.9%)	–
Females [n (%)]	39 (47.6%)	12 (52.2%)	0.69572
Males [n (%)]	43 (52.4%)	11 (47.8%)	
Mean age (years ± SD)	63.2 ± 13.4	65.3 ± 13.5	0.62947
BMI (kg/m^2^ ± SD)	26.7 ± 5.0	26.8 ± 5.0	0.75312
ASA 1 [n (%)]	1 (1.2%)	1 (4.3%)	0.6609
ASA 2 [n (%)]	53 (64.6%)	14 (60.9%)	
ASA 3 [n (%)]	27 (33%)	7 (30.4%)	
ASA 4 [n (%)]	1 (1.2%)	1 (4.3%)	
Any comorbidity [n (%)]	64 (78%)	17 (73.9%)	0.67663
Cardiovascular [n (%)]	30 (36.6%)	8 (34.8%)	0.87442
Hypertension [n (%)]	42 (51.2%)	12 (52.2%)	0.93331
Diabetes [n (%)]	15 (18.3%)	5 (21.7%)	0.71028
Pulmonary disease [n (%)]	6 (7.3%)	3 (13%)	0.38584
Renal disease [n (%)]	6 (7.3%)	2 (8.7%)	0.82658
Liver disease [n (%)]	4 (4.9%)	1 (4.3%)	0.91647
AJCC Stage I [n (%)]	37 (45.1%)	10 (43.5%)	0.63179
AJCC Stage II [n (%)]	24 (29.3%)	5 (21.7%)	
AJCC Stage III [n (%)]	21 (25.6%)	8 (34.8%)	
Colonic resection [n (%)]	53 (64.6%)	16 (69.6%)	0.65748
Rectal resection [n (%)]	29 (35.4%)	7 (30.4%)	
Mean operative time (min ± SD)	194.4 ± 56.7	215.3 ± 73.3	0.44429
Median operative time [min (IQR)]	190 (160–230)	180 (170–275)	
Mean intraoperative blood loss (ml ± SD)	113.0 ± 118.4	128.4 ± 107.6	0.55006
Median intraoperative blood loss [ml (IQR)]	100 (50–150)	100 (50–200)	
Mean length of hospital stay (days, range)	4.8 ± 4.0	10.8 ± 6.7	0.00003
Median length of hospital stay (days, IQR)	4 (3–6)	9 (6–18)	
Readmission [n (%)]	4 (4.9%)	4 (17.4%)	0.04561

The overall infectious complication rate was 21.9%. An analysis of infectious complications is presented in Table 3.

**Table 3:** Types of complications

Anastomotic leakage	9 (8.6%)
Surgical site infection—deep or superficial	6 (5.7%)
Intraperitoneal abscess	2 (1.9%)
Urinary tract infection	3 (2.8%)
Pneumonia	2 (1.9%)
Infectious diarrhoea (C. difficile)	1 (1.0%)

**Fig. 2:**
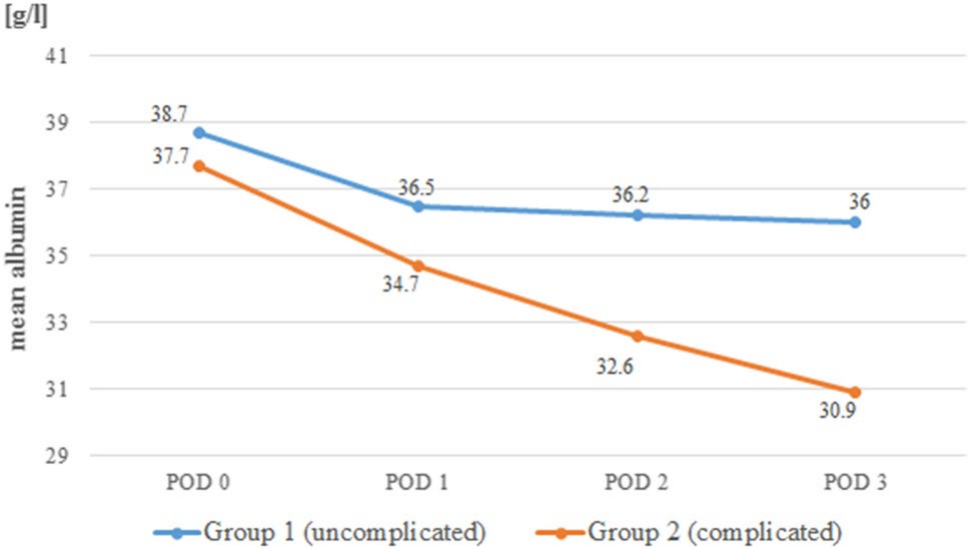
Mean albumin levels in Group 1 and Group 2 in consecutive days

**Fig. 3:**
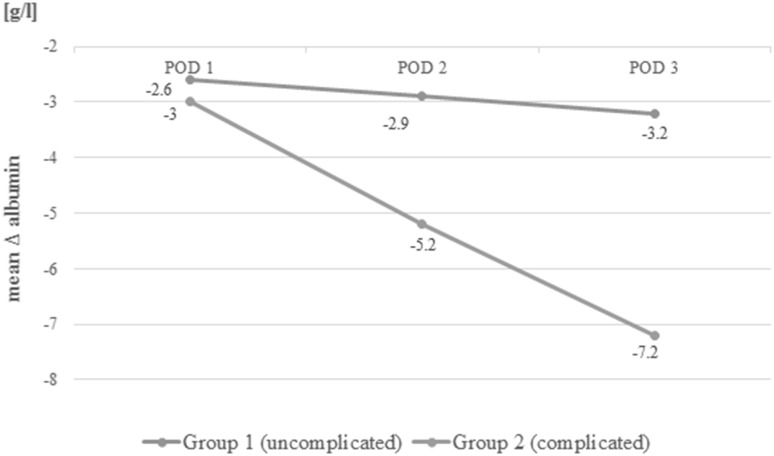
Mean Δ-albumin increments in Group 1 and Group 2 in consecutive days

**Fig. 4:**
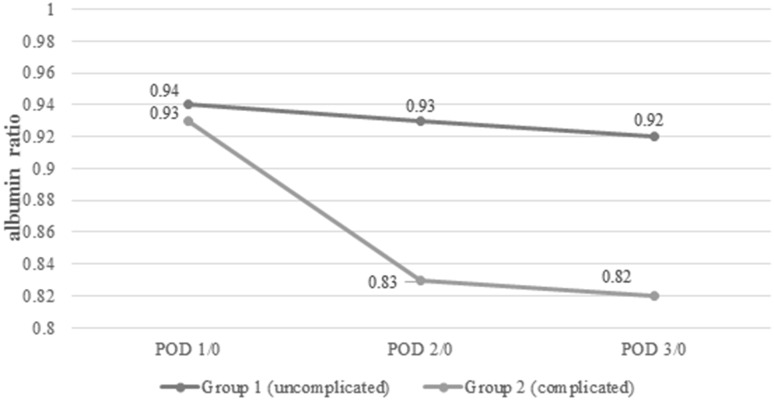
Albumin ratios in Group 1 and Group 2 in consecutive days

Laboratory measurements are presented in Table 4. Before surgery (POD 0 measurement), albumin levels were comparable between groups (*p* = 0.58). On POD 1, the albumin levels decreased in both groups, but the difference between Groups was not statistically significant (*p* = 0.07). On POD 2 and 3, the albumin levels in Group 1 were lower than in Group 2 (*p* = 0.001, *p* = 0.00001) (Fig. 2). Friedman’s ANOVA showed differences in consecutive albumin measurements in both groups (*p* = 0.00012 in Group 1 and *p* < 0.00001 in Group 2). When Δ-albumin increments were analysed, the differences were significant only in Group 2 (*p* = 0.38 in Group 1 and *p* = 0.00006 in Group 2). Similarly, differences in albumin ratios were statistically significant only in Group 2 (*p* = 0.38 in Group 1 and *p* = 0.00006 in Group 2) (Figs.3, 4).

**Table 4:** Analysis of biochemical parameters

Parameter		Group 1 (uncomplicated)	Group 2 (complicated)	*p* value
Mean albumin ± SD (median, IQR) (g/l)	POD 0	38.7 ± 4.9 (39, 36–42)	37.7 ± 5.0 (40, 35–41)	0.58702
	POD 1	36.5 ± 4.2 (37, 34–39)	34.7 ± 4.2 (35, 30–37)	0.07131
	POD 2	36.2 ± 4.1 (37, 34–39)	32.6 ± 5.6 (33, 30–36)	0.00996
	POD 3	36.0 ± 4.4 (36, 34–39)	30.9 ± 3.5 (31, 28–32)	0.00004
Δ-albumin ± SD (median) (g/l)	POD 1	− 2.6 ± 4.1 (− 2, − 5 to 1)	− 3.0 ± 4.6 (− 4, − 6 to 0)	0.68309
	POD 2	− 2.9 ± 4.5 (− 3, − 6 to 0)	− 5.2 ± 5.3 (− 6, − 8 to − 1)	0.04953
	POD 3	− 3.2 ± 4.4 (− 3, − 6 to 0)	− 7.2 ± 4.7 (− 7, − 10 to − 5)	0.00306
Albumin POD 1/POD 0, mean ± SD (median, IQR)		0.94 ± 0.09 (0.94, 0.88–1.02)	0.93 ± 0.13 (0.9, 0.85–1)	0.27379
Albumin POD 2/POD 0, mean ± SD (median, IQR)		0.93 ± 0.11 (0.93, 0.84–1.0)	0.83 ± 0.24 (0.86, 0.79–0.94)	0.02905
Albumin POD 3/POD 0, mean ± SD (median, IQR)		0.92 ± 0.11 (0.91, 0.83–1.0)	0.82 ± 0.11 (0.82, 0.76–0.89)	0.00481
Albumin POD 2/POD 1, mean ± SD (median, IQR)		0.99 ± 0.1 (1, 0.94–1.05)	0.92 ± 0.1 (0.9, 0.86–1.03)	0.01294
Albumin POD 3/POD 1, mean ± SD (median, IQR)		1.0 ± 0.12 (0.97, 0.86–1.03)	0.87 ± 0.08 (0.86, 0.83–0.91)	0.00002

**Fig. 5:**
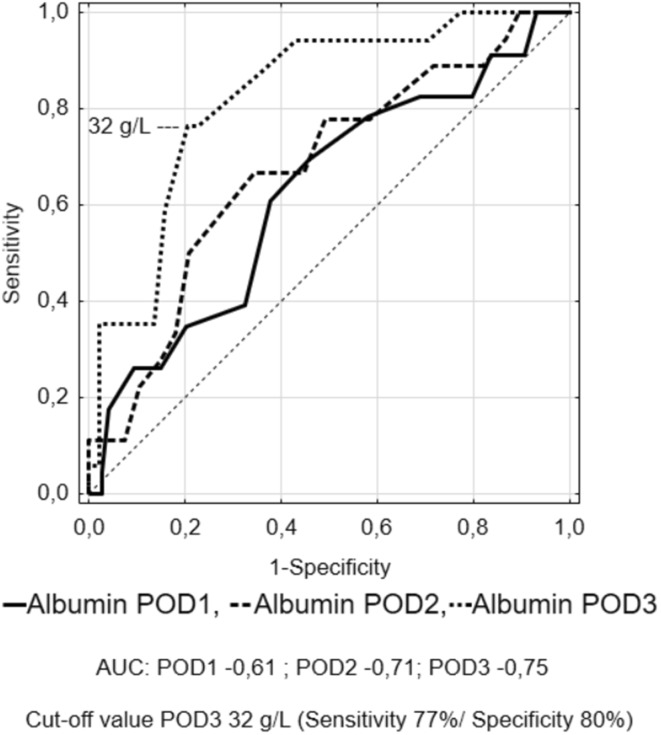
Receiver operating characteristic (ROC) curve to determine the optimal cut-off of albumin measurements

**Fig. 6:**
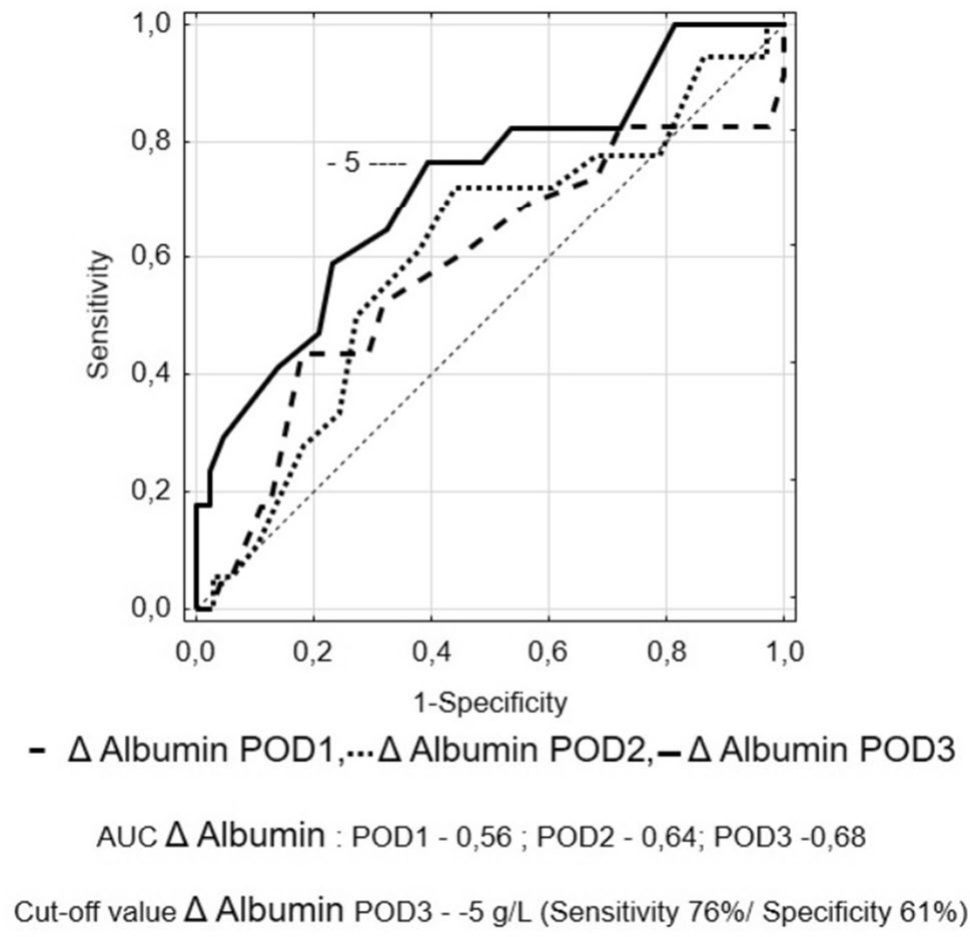
Receiver operating characteristic (ROC) curve to determine the optimal cut-off of Δ-albumin measurements

**Fig. 7:**
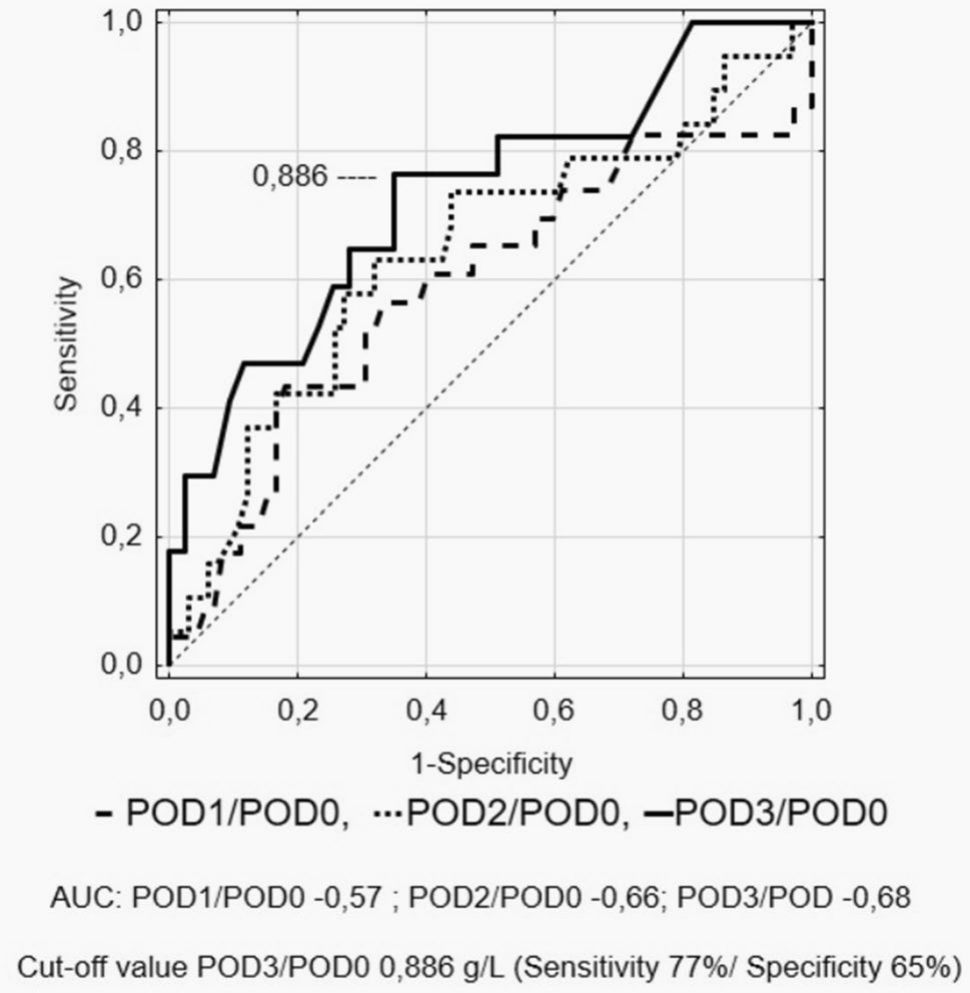
Receiver operating characteristic (ROC) curve to determine the optimal cut-off of albumin ratio measurements (POD0)

**Fig. 8:**
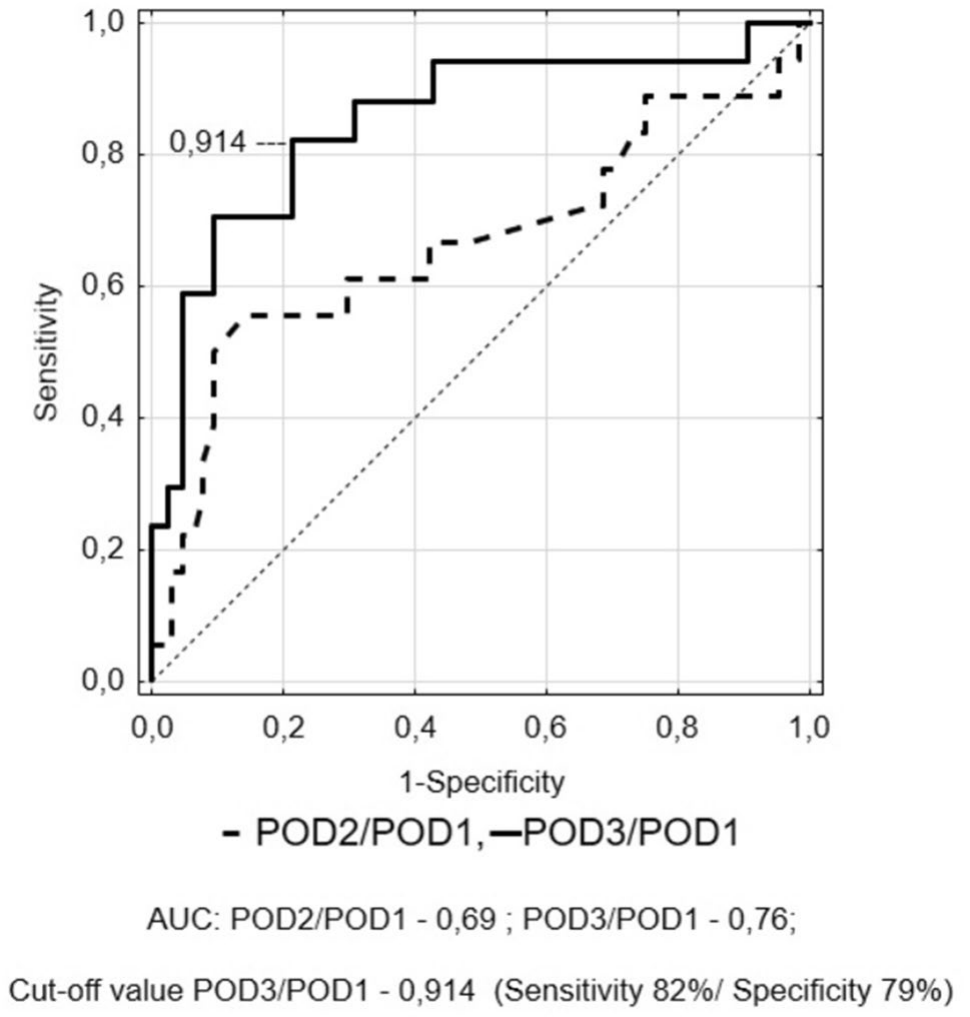
Receiver operating characteristic (ROC) curve to determine the optimal cut-off of albumin ratio measurements (POD1)

A ROC curve was used to determine the optimal cut-off of albumin levels, Δ-albumin increments and albumin ratios in consecutive days. This analysis showed that measurements on POD3 were characterised by highest specificity and sensitivity. Figures 5, 6, 7 and 8 show ROC curves and their characteristics.

## Discussion

This study showed that regardless of the occurrence of complications, there was a reduction in the albumin level in the early PODs after laparoscopic colorectal resection with perioperative ERAS protocol. In addition, it was more pronounced in patients with complications. Moreover, we observed that among uncomplicated cases the level of albumins after initial rapid drop in the first POD remained stable over the next days. In patients who developed complications, albumins decreased further in the consecutive days. That also reflected on the analysis of albumin level derivatives such as Δ-albumin increments and ratios.

Most studies focus on the preoperative level of hypoalbuminemia and its influence on outcomes. Relatively few publications analysed it as a potential marker of early adverse events, and therefore it is seldom used as a biomarker of complications [12, 13, 19, 20]. However, albumin, which is considered a negative acute phase protein with a half-life of 20 days, seems to have perfect characteristics for a useful biomarker [19]. It is easy to measure, widely available and inexpensive. In addition, its kinetics allows for measurements within first postoperative hours [13, 21]. Unfortunately, albumin is rather unspecific, and therefore it cannot exactly predict the underlying cause of its changes.

We focused on albumin, since it is an easy to assess biochemical marker, familiar to most clinicians, and routinely measured in every laboratory. However, apart from albumin, there are other negative acute phase proteins such as transferrin, transthyretin, retinol-binding protein, antithrombin, transcortin, cortisol-binding globulin, transthyretin [22]. Although their decrease is observed in inflammation and they were extensively studied some time ago, according to our knowledge they were never investigated as useful markers of inflammatory complications after surgical procedures. This still needs to be investigated whether other negative acute phase proteins may in the future be used for early detection of complications.

According to previous studies, early postoperative albumin drop is associated with altered metabolism, blood loss, dilution or redistribution into the third space [10, 12, 13, 23]. Albumin production is inhibited in an acute condition, which enables increased production of acute phase proteins such as CRP or fibrinogen. It has been shown in experimental studies that 77% of the postoperative albumin decrease was due to redistribution, while 18 and 6% were attributed to blood loss and catabolism, respectively [10]. Interestingly, redistribution is strongly correlated with systemic inflammatory response observed in major abdominal surgeries and practically non-existent in minor procedures [10, 23].

In contrast to previously published data, rapid postoperative albumin drop in our study was relatively low, 2 g/l (5.1%) in Group 1 and 5 g/l (12.5%) in Group 2. This is different from previously published data, where a 20–35% drop was observed [12, 13]. In these studies, however, the greatest decrease in albumin level was observed in patients undergoing liver surgery [19]. There are two potential factors that might contribute to the diminished albumin drop in our patients. Firstly, all our patients underwent laparoscopic colorectal surgery. It is known that laparoscopy has a positive impact on postoperative stress response, and so may contribute to a decreased albumin drop [24]. In addition, in all our patients ERAS protocol has been used, with a high level of adherence. ERAS has been shown to decrease stress response in the postoperative period [14]. Moreover, one of the key elements of ERAS is balanced/restrictive fluid therapy [25–27]. In our opinion, this might have also prevented excessive hemodilution in the early hours after surgery. All these factors may have an impact on postoperative albumin levels.

The fundamental question is whether the use of albumins in the early postoperative period is clinically relevant. It seems that there is no clear answer to this problem. On the one hand, the kinetics of albumins makes them perfectly suitable as early markers. On the other hand, it drops in any increased stress reaction, making albumins less specific. In addition, their relationship with the extent of surgery, blood loss and fluid resuscitation in the postoperative period can introduce many confounding variables which may bias the interpretation of results. However, we observed that it is not the first drop in the albumin level that differs between complicated and uncomplicated patients but rather the trend over the next days. If the level continues to decrease, it may seem that it is due to an underlying complication, which may require further diagnostics or prolonged observation in hospital.

The ROC curve analysis showed that measurements on POD3 are characterised by the best sensitivity and specificity. Because albumin measurements are rather unspecific in determining the development of complications, we tried to increase the specificity using their derivatives. When Δ-albumin increments were analysed, thus including the baseline levels of albumins in measurements, we observed that the sensitivity and specificity on POD 3 were at the highest level. Similar observations were made when ratios were calculated. Unfortunately, none of these derivatives provided better parameters. In our opinion, it is best to measure albumin levels on consecutive PODs in order to monitor their level when searching for postoperative complications. Other calculations, with the use of albumin levels, do not improve the results.

Our study has certain limitations which are typical for a single centre study. We did not assess food charts and dietetic preferences of patients. They may have introduced confounding factor to the analysis. The study sample is relatively low, especially in the group including patients with complications. Besides, we focused only on infectious complications, since albumin, being the negative acute phase protein, is strongly correlated with inflammatory response. We did not analyse complications with less impact on inflammatory status.

Therefore, our observations should be repeated in larger cohorts of patients. On the other hand, all patients were selected cases, undergoing a similar type of the minimally invasive colorectal procedure. The baseline characteristics of groups of patients with and without complications, as well as the adherence to the protocol were comparable, allowing us to draw the conclusion that the differences are closely related to occurring complications. In addition, types of complications are quite heterogeneous (e.g. surgical site infection vs. anastomotic leakage). The whole idea of the study was based on the influence of inflammatory reaction on changes in albumin levels. Therefore, when planning the study, we decided to focus only on inflammatory complications because albumin, being the negative acute phase protein, is strongly correlated with inflammatory response. We agree that exclusion of other complications (e.g. prolonged ileus) might have influenced the final results. For this reason, it has to be further investigated whether benefits of albumin measurements in the determination of postoperative complications can be found in all patients, regardless of the severity, type and underlying cause of complication.

## Conclusion

Our study showed that a regular measurement of albumin levels in the early postoperative days may be beneficial in the detection of postoperative infectious complications. Although changes in albumins are observed early after surgery, this parameter is relatively unspecific. However, consecutive measurements of albumin levels may very well serve as an auxiliary biomarker in the monitoring of patients after laparoscopic colorectal resections.
